# Bismarck or Beveridge: a beauty contest between dinosaurs

**DOI:** 10.1186/1472-6963-7-94

**Published:** 2007-06-26

**Authors:** Jouke van der Zee, Madelon W Kroneman

**Affiliations:** 1 NIVEL (Netherlands Institute of Health Services Research), P.O. Box 1568, 3500 BN Utrecht, The Netherlands; 2 Faculty of Health Sciences, Department of Medical Sociology, University of Maastricht, Maastricht, The Netherlands

## Abstract

**Background:**

Health systems delivery systems can be divided into two broad categories: National Health Services (NHS) on the one hand and Social Security (based) Health care systems (SSH) on the other hand. Existing literature is inconclusive about which system performs best. In this paper we would like to improve the evidence-base for discussion about pros and cons of NHS-systems versus SSH-system for health outcomes, expenditure and population satisfaction.

**Methods:**

In this study we used time series data for 17 European countries, that were characterized as either NHS or SSH country. We used the following performance indicators: For health outcome: overall mortality rate, infant mortality rate and life expectancy at birth. For health care costs: health care expenditure per capita in pppUS$ and health expenditure as percentage of GDP. Time series dated from 1970 until 2003 or 2004, depending on availability. Sources were OECD health data base 2006 and WHO health for all database 2006. For satisfaction we used the Eurobarometer studies from 1996, 1998 and 1999.

**Results:**

SSH systems perform slightly better on overall mortality rates and life expectancy (after 1980). For infant mortality the rates converged between the two types of systems and since 1980 no differences ceased to exist.

SSH systems are more expensive and NHS systems have a better cost containment. Inhabitants of countries with SSH-systems are on average substantially more satisfied than those in NHS countries.

**Conclusion:**

We concluded that the question 'which type of system performs best' can be answered empirically as far as health outcomes, health care expenditures and patient satisfaction are concerned. Whether this selection of indicators covers all or even most relevant aspects of health system comparison remains to be seen. Perhaps further and more conclusive research into health system related differences in, for instance, equity should be completed before the leading question of this paper can be answered. We do think, however, that this study can form a base for a policy debate on the pros and cons of the existing health care systems in Europe.

## Background

In the domain of health systems research it is not uncommon to divide health systems, or, to be more precise, health care delivery systems into two broad categories: National Health Services (NHS) on the one hand and Social Security (based) Health care systems (SSH) on the other hand, often dubbed after their founding fathers Beveridge (NHS) and Bismarck (SSH) [1-7].

The inclusion of a country's system into one of the two categories is mainly based on the way the systems are funded (general taxation versus earmarked premiums) but these funding differences also correlate with differences in the way the systems are organised (See Table 1).

**Table 1:** Characteristics of the different health care systems

**National Health Services (NHS)** A NHS is funded by means of *general taxation*. *Responsibility for the budget *is in hands of the *Ministry of Health *and as such the NHS is associated with a *strong influence of the state*. The organisation is often part of a *pyramid shaped hierarchical bureaucracy *with primary health care at the bottom and high tech hospitals at the top and goes together with a strict geographic subdivision. *Access to specialized care *is dependent on a referral from a GP: the so-called *gate-keeping system*. Hospitals are state owned and individual GPs have contracts with the NHS. A major *weakness *of the NHS is the *risk for under-funding*. Health care has to compete for public funding with other social segments like education and traffic.
**Social Security Health care system** A SSH is funded by means of *earmarked premiums*, mainly from salaried employees. The system is more loosely organised, with *less state influence and more pluralistic*, with a strong influence of health care providers and (social) insurers. There is often *parallel access *to primary and specialised care and no strict geographic subdivision. Care is provided by *non-profit hospitals and individual practitioners*. Major *weakness *of the system is the *lack of a power centre, cost control is difficult*.

This subdivision into two big groups covers mainly all (West-) European health care systems.

Many aspects of both categories of systems have been studied and described [8-21], but one would have expected that the crucial question: which system is best, which should be preferred, would have been a 'Leitmotiv' in the wealth of studies (see also [22]).

This is not the case, however: the question, in its primitive or in a more sophisticated (that is: by specifying the criteria of comparison) form has rarely been posed and, if so, the answer was mostly inconclusive.

In spite of this general lack of discussion, some authors touched the subject. Firstly, a decade ago, in 1996, Javier Elola (Spain) published in the International Journal of Health Services a paper comparing NHS- and SSH-systems on: health outcomes, health care costs and expenditures and population satisfaction [23]. Using 1992-data he did not find differences in health outcomes between both systems, lower health care costs and better cost-containment in NHS-systems and higher population satisfaction in SSH-systems. Elola used straightforward and overall accepted indicators (infant mortality, life expectancy, potential years of life lost, health care expenditures as % of GDP and per capita, and, for a subset of countries, an indicator of satisfaction with the health care system). A (minor) point of critique could be that he used data at one point in time (1992). He pointed to the trade-off of consumer-satisfaction (SSH-systems) on the one hand and efficiency on the other hand (NHS-systems). Elola called the overcoming of this trade-off of outcomes between the two types of systems a main goal of health care reforms. [23]

Secondly, about a decade later, Saltman and Figueras [1] devote in their book on 'Social health insurance systems in western Europe' a full chapter (60 pages, chapter 4 [24]) to the comparison of SSH and NHS on a wide range of criteria varying from life expectancy, user satisfaction, waiting lists, health care expenditures, fairness in financing, quality ratings etcetera, etcetera. The authors conclude that the relationships (between type of system and criterion) vary 'depending on the parameter of performance being assessed'. They do not find differences in health outcomes between SSH systems and what they call 'northern tax funded countries', a subgroup within the NHS category; they find slightly worse results for 'equity' (mostly funding indicators) in SSH systems and higher population satisfaction rates in the SSH-group. Although the authors cannot provide a clear conclusion, which, in our opinion, is due to the multitude of indicators they used, they end with an extremely relevant policy statement: 'do the higher costs of SSH-systems outweigh the higher population satisfaction given the lack of differences in health outcomes'. (Figueras et al, 2004, p. 133 [24]).

Elola, on his turn, may have played down the importance of his results, because in the abstract of his paper he seemed to recoil from a possible consequence of his study: the return of Southern European countries, that introduced NHS-system in the late seventies and eighties of the 20^th ^century, to their social security roots ([23], p. 239).

Anyway, Elola's paper did not leave a trace in the health systems literature, although it would have formed a very good base for a serious policy discussion about the most desirable direction health care systems should move.

So, we dispose of two sources, a decade apart, in which NHS-systems are compared to SSH-systems. The oldest study seems to yield clear cut results: NHS-systems are cheaper and are better in cost control, and SSH-systems seem to have (differences could not be tested statistically) a stronger public support. There is no difference in health outcomes. The results are valid for 1992. The most recent study concludes firstly that the two types of system do not seem to differ in health outcomes, but that this depends on the indicators used and that there probably is a trade off between health care costs and population satisfaction, but due to a low number of observations (caused by a subdivision of the groups) and a confusingly high number of indicators, the conclusion remains tentative.

### Research problem

In this paper we would like to improve the evidence-base for discussion about pros and cons of NHS-systems versus SSH-systems by adopting Elola's approach using a set of well accepted general performance indicators and testing the robustness of Elola's findings by using time series data instead of a single point in time.

We have the following research questions:

1) Are there indeed no systematic differences in health outcome between NHS- and SSH-systems over a longer period in time (1970–2003)?

2) Do NHS-systems indeed spend less on health care as % of GDP and per capita and are they better in cost control over the same period in time?

3) Is, indeed, the population in SSH-systems more positive about its health care system than in NHS-systems?

4) If differences exist, do they converge over time?

## Methods

### Countries

In this study we restrict ourselves to the Western European countries, where the systems exist over a longer time period and time series analysis is possible. In the study period (1970–2003) some transitions from one system to another have taken place. Greece (1983), Italy (1978), Portugal (1979) and Spain (1986) changed from a SSH system to a NHS system. We deviate from the Saltman and Figueras study in the sense that we excluded Israel (as non-European country) in our study. This results in the following countries (see Table 2):

**Table 2:** Division of countries included in this study in SSH and NHS system^1)^

**Countries with SSH system**	**Countries with NHS system**
■ Austria	■ Denmark
■ Belgium	■ Finland
■ France	■ *Greece (from 1983)*
■ Germany	■ Ireland
■ *Greece (until 1982)*	■ *Italy (from 1978)*
■ *Italy (until 1977)*	■ Norway
■ Luxembourg	■ *Portugal (from 1979)*
■ Netherlands	■ *Spain (from 1986)*
■ *Portugal (until 1978)*	■ Sweden
■ *Spain (until 1985)*	■ United Kingdom
■ Switzerland	

1) The division into SSH and NHS for Greece, Italy, Portugal and Spain is based on the formal introduction of the system, as described in the country descriptions of the Health care system in transition series of the European Observatory in the chapter 'historical background'. [35-40]

### Indicators

The performance indicators for both systems were chosen, based on the study of Elola. These indicators have been collected for each country over a long time period, except for the satisfaction indicator. The performance indicators can roughly be divided into three categories: health outcome indicators, economic indicators (the costs of the system) and the evaluation of the system by the population (satisfaction).

For *health outcome *we used overall mortality rate (standardized death rate per 100,000 inhabitants for all causes), infant mortality rate, and life expectancy at birth.

The *cost *of a country's health care system can be expressed as the cost per head of the population. To compare the costs between countries, these costs are expressed in ppp US$ (power purchasing parities US$). Besides this, the share of health care costs in the total GDP is used. We used both the absolute share as well as the change in share (indexed for the year 2000 = 100).

For *satisfaction *the satisfaction of the population with the health care system as a whole was used for the years 1996, 1998 and 1999.

### Data sources

For each indicator, we used time series from 1970 until 2003 or 2004, depending on the availability of the data.

The health indicators are retrieved from the WHO- Health For All database (standardized death rates per 100,000 for all causes, life expectancy at birth) and OECD health data files 2006 (infant mortality: deaths per 1,000 life births).

The economic indicators are based on the OECD health data files.

Satisfaction with the health care system is based on the indicators that Saltman and Figueras used. Satisfaction data were available for the following countries: Austria, Belgium, Denmark, Finland, France, Germany, Greece, Ireland, Italy, Luxembourg, Netherlands, Portugal, Spain, Sweden, United Kingdom. Data for 1996, 1998 and 1999 are based on the results of questions concerning the satisfaction with the (organisation) of the health services in the countries concerned from the Eurobarometer studies (Eurobarometer 44.3 (1996), 49 (1998), and 52.1 (1999)).

### Analyses

For each indicator, time series will be displayed for each country, with (unweighted) averages calculated for each system and we will discuss the differences between these averages.

### Ethical approval

Ethical approval was not required for this study.

## Results

### Health outcome indicators

#### - Overall (age standardized) mortality rates

**Figure 1:**
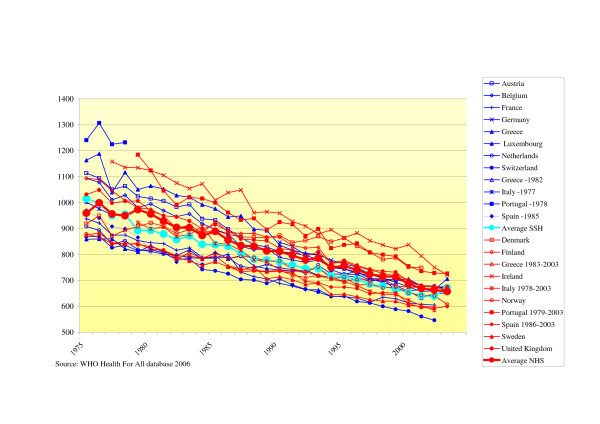
Standardized death rates per 100,000 inhabitants for all causes.

Mortality rates were declining over the years irrespective of the health care system. However, there is no convergence: the range between minimum and maximum mortality rates hardly changed. There is a small difference in average mortality rates between NHS and SSH, in favour of the SSH. The SSH had on average over the years a 5% lower mortality rate (see Fig. 1).

#### - Infant mortality rate

**Figure 2:**
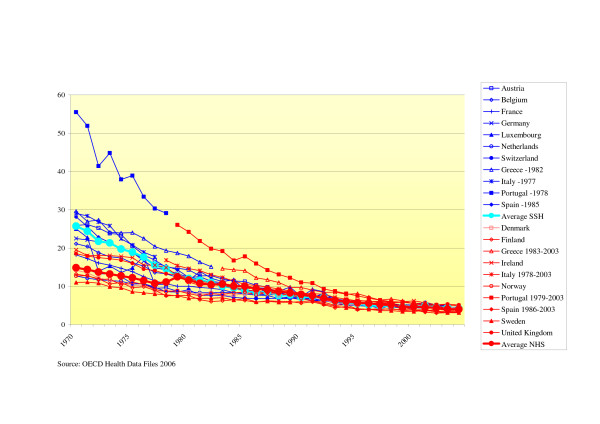
Infant mortality rates (deaths per 1.000 life births).

There has been a clear converging trend in infant mortality rates. Differences between countries became rapidly smaller over the years. We found that until 1982, NHS had lower rates and from 1983 until 1998, SSH had a (on average 6%) lower infant mortality rate, although the differences were much smaller compared to the previous period. From 1999 until 2004, the differences between NHS and SSH become negligible (see Fig. 2).

#### - Life expectancy

**Figure 3:**
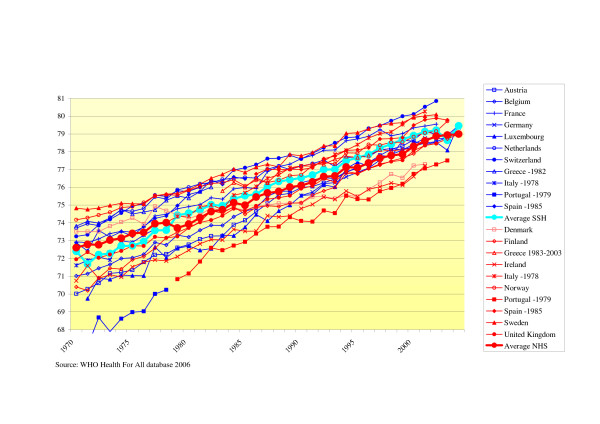
Life expectancy at birth.

Life expectancy increased over the years. From 1970–1985 there is convergence between both groups of systems from 1985 onwards; the range between minimum and maximum life expectancy did not change. In the 1970s, life expectancy was higher in NHS-countries (approximately 0.5 years), in the later years (1980–2002) SSH had a slightly higher life expectancy (about 0.5 years) (see Fig. 3).

### Health care expenditure indicators

#### - Health expenditure per capita

**Figure 4:**
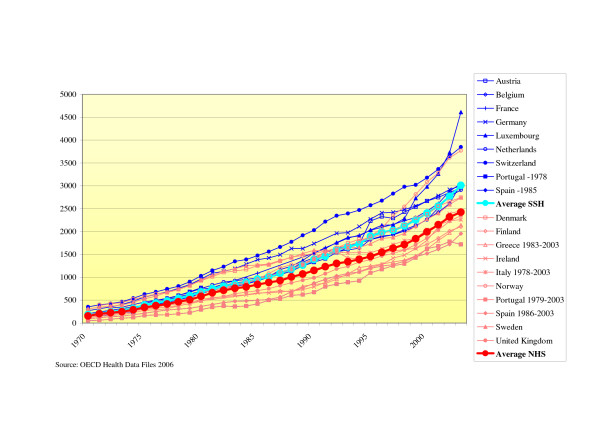
Total health care expenditure per capita (PPP-US$).

The expenditure per capita showed a diverging trend. In SSH systems, the expenditure on health per capita has become increasingly higher compared to NHS-systems (see Fig. 4).

#### - Health care expenditure as percentage of GDP

**Figure 5:**
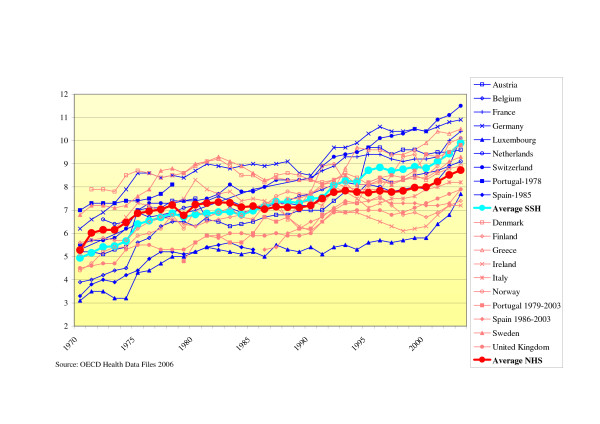
Total health care expenditure as percentage of GDP.

The share of health care expenditures in GDP is increasing in the 1970s for both systems and in the 1980s, cost containment methods seem to be effective in both systems, since the share of health care expenditure is more or less constant. From 1993, we see an increase again for the SSH systems, the NHS-systems follow at a lower pace (see Fig. 5). Over time, in SSH systems the share of health care expenditure in GDP increased from 5% in 1970 to 10% in 2003. The NHS systems increased on average from 5% to 8% in the same period.

### Satisfaction with the health care system

**Figure 6:**
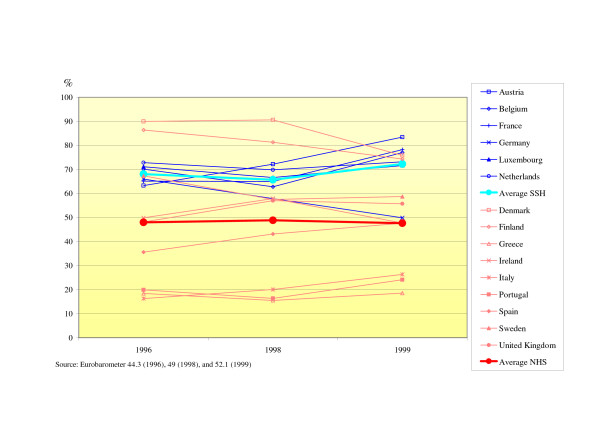
Satisfaction with health care system (% of population very or fairly satisfied).

The satisfaction with the health care system of the population in SSH-countries is much higher compared to NHS countries (see Fig. 6). In SSH countries about two-third of the population is very or fairly satisfied with the system, whereas in NHS countries this is the case for only half of the population. Within NHS countries, the variation is substantial. Denmark and Finland show even higher satisfaction rates compared to all SSH countries in 1996 and 1998. The satisfaction within SSH countries showed less variation. The only SSH country that showed a decrease in satisfaction rates is Germany. The individual NHS countries showed a converging trend over the three years towards the mean.

## Discussion

Our first research question concerned differences in health outcome between NHS and SSH systems over time. Our study revealed a strong improvement in life expectancy and a reduction in infant mortality regardless of the system. In our time series, since the 1980s, SSH systems showed slightly favourable mortality rates compared to NHS systems, a persistent difference that continued to exist over time. Also for the life expectancy at birth, SSH systems persistently performed better than NHS systems since the 1980s, although the differences are small. Infant mortality rates showed a converging trend without any difference between both systems since the beginning of the 1980s. Therefore, the conclusion of both Elola and Figueras et al, based on cross sectional data, that there were no differences in health outcomes between NHS and SSH systems is not fully supported by our study.

Our second research question concerned the differences in health care expenditure. The costs of NHS systems are persistently lower compared to SSH systems over time both in terms of health expenditure per capita and as percentage of GDP (although the latter is the case only since 1985, before this time NHS systems were consuming a larger part of the GDP). So, both Elola's and Figueras and Saltman's conclusion that NHS systems perform better in controlling costs is supported by our study.

The third research question was: is the population in SSH systems more positive about its health care system than in NHS systems. Elola's conclusion that SSH systems receive greater public support is also supported by our study.

The last research question concerned a possible convergence of the systems over time. The results of our study show that, except for infant mortality rates, this convergence did not take place yet. This is not in line with the hypothesis of Elola, who argued that health care reforms were directed towards convergence between both systems.

Of course this study has several limitations, that we would like to discuss here. Firstly we will address the pitfalls of international comparative research, secondly the effect of the small numbers of countries and thirdly the consideration whether we used the right set of outcome indicators.

### Pitfalls of international comparisons

International comparison of health data is severely hampered by differences in national definitions and differences in national methods of data collection [22,25,26]. Mosseveld, in a thesis on international comparison of health accounts, argued that the analysis of time trends is to be preferred to cross-sectional comparisons [25]. To our opinion, the advantage of time series in this study was that relatively small differences that may not have been noted in cross-sectional analyses, appeared to be quite persistent over time, thus contributing to the opinion that the differences are structural in nature.

### Small numbers

Due to the small numbers of countries it is possible that a country with extreme values influences the results strongly. In our study this could have been the case with Portugal. The health indicators in Portugal were inferior to all other countries, although a spectacular improvement could be observed over time. From 1985 onwards, Portugal is performing at a comparable level with other relatively low performing countries in our study, but before 1985, Portugal was performing worse. Portugal had a SSH until 1978 and a NHS from 1979. Portugal's switch might have influenced the average of both groups considerably, that is to say until 1985. Elola excluded Portugal from the analyses because he argued that although Portugal formally had changed towards an NHS the practical implementation was incomplete: parts of the SSH system continued to exist. However, the same could be concluded for Greece, where also parts of the SSH system continued to exist after the formal change towards a NHS system. Since none of the countries in our study have a pure NHS or SSH system anyway and formulating criteria for including and excluding a country into one of both systems will be always disputable, we decided to opt for the formal introduction of the law concerning the organisation of the health care system as criterion for classifying the country's health care system [27]. The exclusion of Portugal from our analysis, however, did not change the results, although the differences in health outcomes became somewhat smaller. Exclusion of the 'switchers' (that is, countries that changed from SSH to NHS in the study period), only influenced the outcome on satisfaction, since three of the four 'switchers' had very low satisfaction rates. However, data on satisfaction are available for a very small time period only and this time period is at least a decade after the switch, so it is very improbable that the switch as such was the cause of the low satisfaction rates.

### Did we use the right (outcome) indicators ?

The health indicators used in this study are relatively basic indicators. The advantage of these basic indicators is that their definition is relatively stable over time and among countries. A more sophisticated health indicator is the (recently introduced) Health Adjusted Life Expectancy (HALE).We analysed the differences in HALE between the two groups; SSH countries had a slightly higher Health Adjusted Life Expectancy than NHS countries. HALE data were however only available for a short period of time (1999–2002) and have also been subject to a change in definition in those years [28]. So, for our purpose, analysing long term differences, HALEs were not suitable.

The stronger public support of SSH-systems is a result that has been found in several studies now [8,23,29]. Dissatisfaction with a health care system seems to be related to problematic organizational items like waiting lists and limitations in accessibility, like the gate-keeping system. In countries where GPs act as gatekeepers, the public was less positive about organisational aspects of primary care; no differences were found in satisfaction of the medical quality and the communication with the patients [8]. The lower satisfaction of the population of NHS systems may be attributed to the existence of waiting lists [24] and limitations in the accessibility of secondary care, as in the gate-keeping system [8,30].

Although health outcomes may be influenced by financial and organisational opportunities within the medical world, this will not be the only contributing factor. For instance, life style factors (e.g. smoking habits, diet, alcohol consumption) contribute largely to health outcomes. However, transforming life style factors into more healthy habits can be reached more effectively outside the health care system.

### Equity

One might argue that the indicators selected in this study do not cover all relevant criteria in health care systems analysis. Equity, for instance, is an overall criterion that forms the corner stone of the National Health Service. Comparing NHS and SSH systems without taking into account the concept of equity might be considered as not completely fair. The point is, however: what is equity and is it an unequivocal concept?

Equity in health care can be described as follows: those with equal needs should be equally treated and those with greater needs should receive greater attention and more resources [31]. Mayberry e.a. [32] distinguished three dimensions of equity: access, use and outcomes. In addition, Van Doorslaer and Wagstaff e.a. [20,33] distinguished another dimension: the distribution of the financial burden of the health care system. In the Health Services Research Community the debate on the research agenda concerning equity is currently going on and certainly not yet concluded [32,34].

## Conclusion

We think that our study can form a base for a policy debate on the pros and cons of the existing health care systems in Europe as far as health outcomes, health care expenditures and patient satisfaction are concerned. For the issue of equity firstly a discussion is needed on what indicators are relevant and secondly, more research is needed into these indicators. The debate on 'which system is best' should take place at national level and at European level. The big challenge is to reconcile organisational restrictions like waiting lists and gate-keeping with consumer preferences. Countries that combine high satisfaction rates with organisational restrictions (like Denmark, Finland and the Netherlands) could form examples for their neighbours. Further more, the results of our study could contribute to the discussion for the choice of health care systems in countries that are in the process of implementing (universal) health care insurance, like middle income countries or the newly independent states of the former Russian Federation.

## Abbreviations

GDP Gross Domestic Product

NHS National Health Services

SSH Social Security Health care system

## Competing interests

The author(s) declare that they have no competing interests.

## Authors' contributions

JvdZ initiated the study and drafted the manuscript. MK participated in the design of the study, the analyses of the data and helped to draft the manuscript. Both authors read and approved the final manuscript

## Pre-publication history

The pre-publication history for this paper can be accessed here:


